# Role of copper homeostasis and cuproptosis in heart failure pathogenesis: implications for therapeutic strategies

**DOI:** 10.3389/fphar.2024.1527901

**Published:** 2025-01-09

**Authors:** Zhichao Liu, Yongkang Gan, Zhen Shen, Siqi Cai, Xizhen Wang, Yong Li, Xiaofeng Li, Huanjie Fu, Jinhong Chen, Ningcen Li

**Affiliations:** ^1^ School of Rehabilitation Medicine, Shandong Second Medical University, Weifang, Shandong, China; ^2^ Department of Vascular Surgery, Tianjin Academy of Traditional Chinese Medicine Affiliated Hospital, Tianjin, China; ^3^ Department of Clinical Laboratory, Affiliated Hospital of Shandong Second Medical University, Weifang, Shandong, China; ^4^ College of Art, Nanjing University of Information Science and Technology, Nanjing, Jiangsu, China; ^5^ Experimental Center for Medical Research, Shandong Second Medical University, Weifang, Shandong, China; ^6^ Department of Cardiovascular, Second Teaching Hospital of Tianjin University of Traditional Chinese Medicine, Tianjin, China; ^7^ Research Center of Experimental Acupuncture Science, Tianjin University of Traditional Chinese Medicine, Tianjin, China

**Keywords:** copper, copper homeostasis, cuproptosis, mitochondrion, heart failure

## Abstract

Copper is an essential micronutrient involved in various physiological processes in various cell types. Consequently, dysregulation of copper homeostasis—either excessive or deficient—can lead to pathological changes, such as heart failure (HF). Recently, a new type of copper-dependent cell death known as cuproptosis has drawn increasing attention to the impact of copper dyshomeostasis on HF. Notably, copper dyshomeostasis was associated with the occurrence of HF. Hence, this review aimed to investigate the biological processes involved in copper uptake, transport, excretion, and storage at both the cellular and systemic levels in terms of cuproptosis and HF, along with the underlying mechanisms of action. Additionally, the role of cuproptosis and its related mitochondrial dysfunction in HF pathogenesis was analyzed. Finally, we reviewed the therapeutic potential of current drugs that target copper metabolism for treating HF. Overall, the conclusions of this review revealed the therapeutic potential of copper-based therapies that target cuproptosis for the development of strategies for the treatment of HF.

## 1 Introduction

Heart failure (HF) is one of the most prevalent cardiovascular diseases worldwide and poses a significant threat to human health (Zannad, 2018). It is characterized by impaired cardiac function due to ventricular filling and ejection dysfunction and represents the end stage of various cardiovascular disorders (Ziaeian and Fonarow, 2016). Despite ongoing advancements in treatment methods, the prognosis for patients with HF remains poor, making it a serious public health concern (Ambrosy et al., 2014). Furthermore, HF places a substantial economic burden on healthcare systems (Ziaeian and Fonarow, 2016). Consequently, a thorough understanding of the potential therapeutic targets and underlying mechanisms of HF is crucial for improving its prognosis and patient outcomes.

Copper is an essential micronutrient and a vital catalytic cofactor involved in various biological processes, including the production of biomolecules, antioxidant defense, and mitochondrial respiration (Chen L. et al., 2021; Maung et al., 2021). Copper homeostasis is tightly regulated, as both excessive and deficient levels of copper can lead to pathological changes that adversely affect human health (Maung et al., 2021). Dysregulation of copper homeostasis can contribute to the pathogenic mechanisms of HF by influencing inflammation (Wang et al., 2022), oxidative stress (Tsutsui et al., 2011; Vo et al., 2020), energy metabolism (Fan et al., 2005), cell death (Vanempel et al., 2005; Jiang et al., 2022), responses to β-adrenergic stimulation (Elsherif et al., 2004b), and calcium homeostasis (Elsherif et al., 2007). Additionally, a newly identified pattern of copper-dependent cell death, namely, cuproptosis, may facilitate the occurrence of HF by affecting mitochondrial function.

This review aimed to investigate the roles of copper homeostasis and cuproptosis in HF, highlighting their potential for the development of therapeutic strategies for HF by targeting cuproptosis. The conclusions of this review may provide insights into future research directions regarding the relationship between cuproptosis and HF.

## 2 Copper homeostasis biochemical and molecular insights

### 2.1 Systemic copper metabolism

**FIGURE 1:**
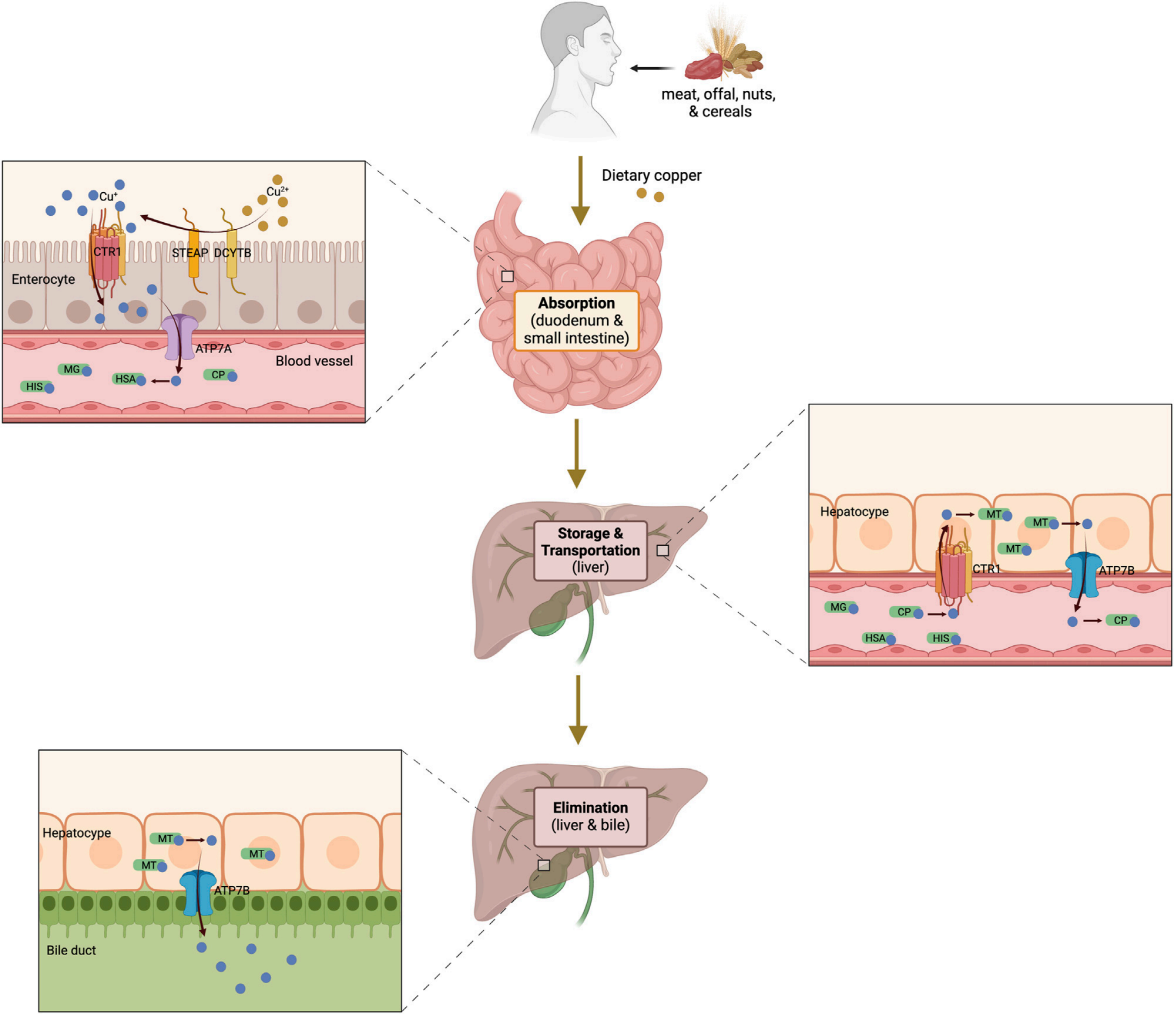
Systemic copper metabolism pathway diagram. Dietary copper absorption occurs primarily in the duodenum and small intestine. Cu^2+^ is reduced to Cu⁺ by STEAP and DCYTB, after which Cu^+^ enters enterocytes via CTR1. Subsequently, ATP7A facilitates its transport and release into the circulation, where it binds soluble chaperones and is transported to the liver through the portal vein for storage and further transportation. Excess copper is excreted into the bile by the liver. The figure was created using BioRender. CTR1, copper transporter 1; STEAP, six-transmembrane epithelial antigen of the prostate; DCYTB, duodenal cytochrome b; ATP7A and 7B, ATPase copper transporter 7A and 7B; CP, ceruloplasmin; MG; macroglobulin; HAS; human serum albumin; HIS, histidine; MT, metallothionein.

In the field of “metals in biology,” metals play unique and crucial roles in biological systems. Copper is an essential trace metal found in nearly every living organism, with a normal human body containing approximately about 100 mg (Wang D. et al., 2023). It is primarily found in muscles, bones, and the liver, with small quantities present in the blood (Festa and Thiele, 2011). Copper exists in two distinct ionic forms, namely, cuprous ions (Cu [I], reduced type) and cupric ions (Cu [II], oxidized type), both of which are involved in the enzymatic modulation of cellular physiological activities. However, redox cycling between Cu(I) and Cu(II) can contribute to the catalytic generation of highly toxic hydroxyl radicals, subsequently damaging macromolecules (Halliwell and Gutteridge, 1984). Therefore, it is necessary to maintain systemic copper levels within a stable range to ensure proper biochemical processes and prevent cellular damage (Figure 1).

#### 2.1.1 Copper uptake

Copper is acquired primarily from dietary sources such as meat, offal, nuts, and cereals (Bost et al., 2016). The absorption of dietary copper, predominantly in the form of Cu(II), occurs mainly in the small intestine (Mason, 1979) by intestinal epithelial cells and is modulated by copper transporter 1 (CTR1), which is encoded by the solute carrier family 31 member 1 (SLC31A1) gene and located on the apical surface of the cells (Petris, 2004). Reportedly, CTR1 is crucial for absorption, and its systemic or tissue-specific deletion significantly reduces dietary copper absorption (Lee et al., 2002). Additionally, the activities of duodenal cytochrome b (DCYTB) and six-transmembrane epithelial antigen of the prostate (STEAP) facilitate this process by reducing Cu(II) to Cu(I) (Dancis et al., 1992; Georgatsou et al., 1997), the ionic state that is transported by CTR1.

#### 2.1.2 Copper transport and storage

Following copper absorption through intestinal epithelial cells, it is secreted into the bloodstream and binds soluble chaperones such as ceruloplasmin (CP), human serum albumin, macroglobulin, and histidine (Moriya et al., 2008; Ramos et al., 2016; Lutsenko, 2021). These complexes transport copper to the liver via the portal vein, where hepatocytes take up copper through CTR1. Within hepatocytes, copper can either be transported to specific proteins via copper chaperones or chelated by the copper-binding protein metallothionein (MT) for storage (Luza and Speisky, 1996; Krężel and Maret, 2017). Thus, the liver serves as the primary organ responsible for capturing, distributing, and excreting copper, playing a crucial role in regulating systemic copper homeostasis. The ATPase copper transporter 7A (ATP7A) and ATPase copper transporter 7B (ATP7B) mediate copper transport in peripheral and liver tissues, respectively (Wang et al., 2011; Telianidis et al., 2023). ATP7A facilitates the transport of copper to the portal vein, whereas ATP7B is responsible for pumping copper back into the bloodstream from the liver. In the blood, copper ions can again bind soluble chaperones, allowing them to be delivered to various organs and tissues where they catalyze reactions involved in numerous physiological processes (Lutsenko et al., 2007b; La Fontaine et al., 2010).

#### 2.1.3 Copper elimination

Excess endogenous copper is primarily excreted through bile and subsequently eliminated in feces (Turnlund, 1998). Other routes, such as sweat, urine, or menstruation, have a lesser impact on copper excretion or depletion. ATP7B plays a crucial role in removing excess copper from the body, and its inactivation (such as in Wilson’s disease) can lead to copper accumulation in the liver and subsequent copper-induced toxicity (Yang et al., 2023). Notably, the excretion of endogenous copper has been reported to be significantly affected by dietary copper intake (Turnlund et al., 1989; Scott and Turnlund, 1994).

Taken together, the processes of copper absorption, storage, transport, and elimination in organisms determine the distribution and modulation of copper homeostasis in the body.

### 2.2 Copper homeostasis is tightly regulated within cells

**FIGURE 2:**
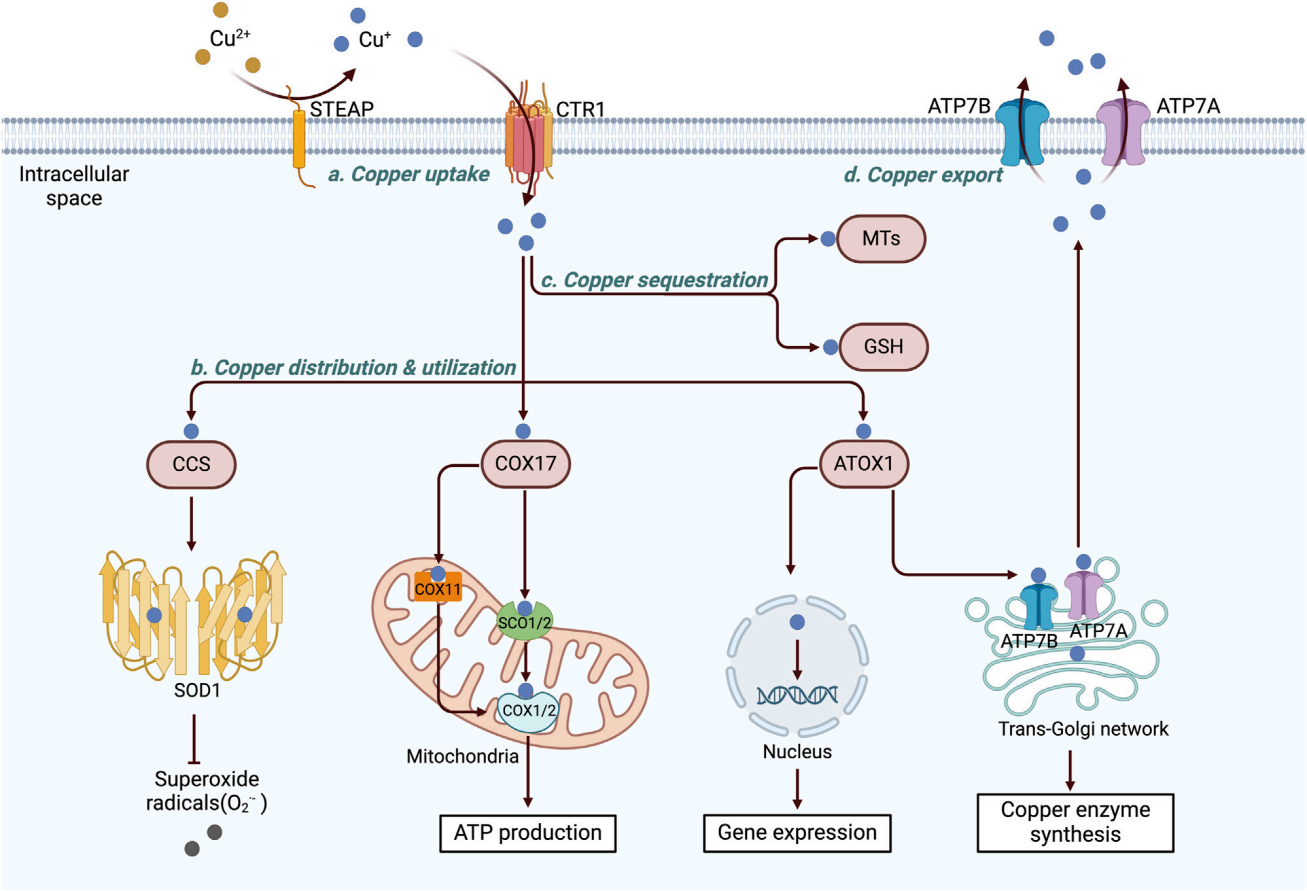
Diagram of the cellular copper metabolism pathway. Within cells, copper ions are delivered to various cellular components, including the cytoplasm, mitochondria, nucleus, and TGN, via a complex, high-affinity copper chaperone system. CCS transports copper to SOD1, where it helps mitigate oxidative stress. COX17 directs copper to the mitochondria, where it is used by COX to activate enzymes in the respiratory chain. ATOX1 transfers copper to the nucleus, where it binds to transcription factors and drives gene expression. Additionally, ATOX1 facilitates copper transport to the trans-Golgi network, promoting the synthesis of copper-dependent enzymes. Excess intracellular copper is sequestered by two key molecules, MT and GSH. Copper is then exported from the cell by ATP7A/B, which relocates copper from the TGN to the plasma membrane, where it is pumped into the extracellular space via exocytosis. The figure was created using BioRender. CTR1, copper transporter 1; STEAP, six-transmembrane epithelial antigen of the prostate; CCS, copper chaperone for superoxide dismutase; SOD1, superoxide dismutase 1; COX17, cytochrome c oxidase copper chaperone 17; cytochrome c oxidase copper chaperone 11 (COX11); SCO1/2, synthesis of cytochrome c oxidase 1/2; COX, cytochrome c oxidase; ATOX1, antioxidant 1 copper chaperone; ATP7A and 7B, ATPase copper transporters 7A and 7B; MT, metallothionein; GSH, glutathione.

Copper is essential for cellular activity, and its intracellular concentration is meticulously regulated to prevent the detrimental effects of both copper deficiency and overload (Dupont et al., 2011; Kim et al., 2013). Copper homeostasis and compartmentalization are mediated by a finely tuned network of copper transport proteins, soluble chaperones, copper enzymes, and copper-dependent transcriptional regulators (Burkhead et al., 2009; Robinson and Winge, 2010; Festa and Thiele, 2011). The levels of copper are maintained within a narrow range through the synergistic actions of these copper-dependent proteins (Figure 2).

#### 2.2.1 Copper absorption

The high-affinity copper transporter CTR1 (encoded by *SLC31A1*) is a transmembrane protein that forms a stable trimeric channel (Ren et al., 2001), facilitating the absorption of most copper ions in cells by allowing their passage across the plasma membrane (Maryon et al., 2013; Bian et al., 2023). *In vitro* studies have shown that the expression of CTR1 can be modulated in a copper-dependent manner and that it is upregulated under copper-depleted conditions to increase copper uptake and downregulated under copper-overloaded conditions to prevent copper cytotoxicity (Liang et al., 2012; Maryon et al., 2013). CTR1 plays a critical role in maintaining copper homeostasis.

#### 2.2.2 Intracellular copper distribution and utilization

Copper is utilized in various cellular compartments, and its intracellular distribution varies according to metabolic requirements (Lutsenko, 2021). Once copper enters the cell, it is allocated by copper chaperones to specific protein targets within intracellular structures such as the trans-Golgi network (TGN), mitochondria, or nucleus. Three main copper chaperones have been identified within mammalian cells: antioxidant 1 copper chaperone (ATOX1), cytochrome c oxidase (COX) copper chaperone 17 (COX17), and copper chaperone for superoxide dismutase (CCS) (Finney and O’Halloran, 2003; Banci et al., 2010).

ATOX1 can deliver copper to ATP7A and ATP7B within the TGN, facilitating the synthesis of copper-dependent enzymes such as lysyl oxidase (LOX), CP, and tyrosinase (Petris et al., 2000; Hellman et al., 2002; Shanbhag et al., 2019). Additionally, ATOX1 functions as a copper-dependent transcription factor, transporting copper into the nucleus and promoting cell proliferation (Itoh et al., 2008). It collaborates with specificity protein 1 and metal-regulatory transcription factor 1 to regulate copper-dependent gene expression (Selvaraj et al., 2005; Liang et al., 2012).

CCS transfers copper to superoxide dismutase 1 (SOD1), aiding in the detoxification of reactive oxygen species (ROS) while maintaining copper homeostasis (Rae et al., 2024). The metabolism of oxygen in mitochondria is associated with the production of superoxide, which damages cells. SOD1 functions as a key antioxidant enzyme, converting superoxide radicals into molecular oxygen and hydrogen peroxide (Leitch et al., 2009). CCS and SOD1 are colocalized and interact within various cell types (Casareno et al., 2024; Rae et al., 2024; Rothstein et al., 2024); however, the exact mechanism of their concurrent transportation to the mitochondria remains unclear (Suzuki et al., 2000).

COX17 transports copper from the cytosol to the mitochondrial inner membrane, facilitating the synthesis of COX (SCO)1 and SCO2 and promoting the insertion of copper into the mitochondrially encoded COX subunit 2 (COX2) (Stiburek et al., 2024). Additionally, copper can be transported via COX17 to COX1 from the cytoplasm through COX 11 (COX11) (Hiser et al., 2000). Both COX1 and COX2 contain redox-active copper centers that play crucial roles in electron transfer within complex IV, ultimately promoting ATP generation (Nývltová et al., 2000). Mutations in SCO1, SCO2, and COX17 are associated with reduced COX activity, leading to mitochondrial dysfunction and oxidative stress (Takahashi et al., 2002; Leary et al., 2004).

#### 2.2.3 Intracellular sequestration

Within cells, labile copper can produce ROS and cause cytotoxicity. This harmful process is effectively mitigated by proteins that sequester intracellular Cu(I). Specifically, excessive intracellular copper is chelated by two main antioxidant peptides: glutathione (GSH) and MTs (Shishido et al., 2001; Aliaga et al., 2016). Additionally, copper is stored in specialized vesicles and secretory granules (Bonnemaison et al., 2009; Leary and Ralle, 2020).

Emerging evidence indicates that the thioredoxin system contributes to the regulation of copper-induced oxidative stress, particularly in neuronal cells (Tanaka et al., 2018). Thioredoxin-albumin fusion proteins have been shown to suppress ROS production and downregulate oxidative stress-related gene expression without significantly affecting intracellular copper levels (Tanaka et al., 2018). These findings suggest an antioxidative mechanism independent of direct copper sequestration, which may have broader implications for mitigating copper-mediated cytotoxicity in other tissues, including cardiac cells.

#### 2.2.4 Copper export

The export of intracellular copper relies on transporting proteins capable of actively removing excessive copper. In humans, ATP7A and ATP7B are critical copper transporters (La Fontaine and Mercer, 2007). When cellular copper levels are increased, these transporters undergo copper-mediated conformational changes and translocate from the TGN to the cell membrane, thereby promoting copper transport (Petris and Mercer, 1999; Yang et al., 2023). This process requires energy from ATP hydrolysis to transport copper along a concentration gradient (Lutsenko, 2021). Therefore, the activities and trafficking of ATP7A and ATP7B are stringently controlled by intracellular copper concentrations, copper-binding proteins such as MTs, and multiple signaling pathways (La Fontaine and Mercer, 2007; Lutsenko et al., 2007a; Gupta and Lutsenko, 2009).

## 3 Evidence linking copper dyshomeostasis to HF

Any abnormality or combination of abnormalities that disrupt cardiac structure, mechanics, or electrical function can potentially trigger HF. Conditions such as atherosclerosis, myocardial infarction (MI), cardiomyopathy, hypertension, and valvular heart disease (VHD) are notable contributors (Heidenreich et al., 2022). Among these, ischemic heart diseases have a significant effect on both acute and chronic HF(Arrigo et al., 2020). Research has increasingly linked the dysregulation of copper homeostasis—resulting in either excessive or deficient copper levels—to the development of HF.

Higher copper levels have been extensively suggested to be associated with HF (Table 1). For example, several prospective cohort studies have indicated that increased serum copper levels are significantly linked to increased HF-related mortality (Málek et al., 2003; Malek et al., 2006). An increased serum copper-to-zinc ratio has also been associated with increased HF risk among middle-aged Finnish males (Kunutsor et al., 2022). Additionally, Hammadah et al. reported in a study involving 890 patients who higher levels of CP, which transports over 95% of copper in the body, were related to an increased risk of HF and poor prognostic outcomes (Hammadah et al., 2014). Measuring CP levels in conjunction with N-terminal pro-B-type natriuretic peptide levels is advantageous for identifying high-risk HF patients during a 1-year follow-up (Romuk et al., 2004). Further investigations into the relationship between myocardial and serum copper contents in patients with HF are essential. In some experiments, coronary infusion of CuCl_2_ solutions can induce acute cardiac dysfunction, with the effects of Cu(II) infusion occurring within minutes in both diabetic and normal hearts, suggesting that these effects are not due to remodeling (Cheung et al., 2015). These findings suggest that increased copper content may be related to the pathogenic mechanisms underlying HF.

**TABLE 1:** Evidence linking copper excess and HF.

Country, author, year	Methods and study population	Result
Greece, Alexanian et al. (2014)	125 patients with AHF or CHF (71% male, aged 69 ± 11 years) and 21 healthy volunteers matched controls	Serum Copper was increased both in AHF (*p* = 0.006) and CHF (*p* = 0.002) and correlated with left ventricular systolic and diastolic function
India, Singh et al. (1985)	44 cases of AMI and 23 cases of angina and 40 age and sex matched healthy controls; HF was most frequent complication (25%, 11 cases) during 28-day follow-up	Mean peak serum copper levels were higher in cases of AMI with complications (203.15 ± 15.13 μg%) as compared to cases of AMI without complications (170.54 ± 14.18 μg %) (*p* < 0.001). AMI with congestive HF had significantly higher serum copper levels as compared to controls
United Kingdom, Kunutsor et al. (2022)	1,866 men aged 42–61 years; 365 HF cases occurred during 26.5 years median follow-up	The HR (95% CI) for incident HF per unit increase in serum copper was 2.71 (1.50–4.89). As serum copper levels increase, the risk of HF gradually increases
Czech Republic, Malek et al. (2006)	30 consecutive subjects with acute decompensation of CHF and 30 patients with chronic stable HF, follow-up of 12 months	The time to the combined end-point death or hospital admission was significantly affected by serum copper level (19.1 vs. 15.6 μmol/L, *p* < 0.0001). Serum copper levels predicted short term outcome in high risk patients with chronic heart failure
Czech Republic, Málek et al. (2003)	64 patients with CHF; 30 patients died or were admitted to the hospital because of worsening HF during 12 months follow-up	Baseline serum copper concentrations are significantly higher in patients with CHF (*p* < 0.001), also correlating with higher 1-year mortality and morbidity
Iran, Shokrzadeh et al. (2009)	30 ISCMP patients and 27 healthy volunteers	The mean copper level of the ISCMP group (1.54 ± 0.52 mg/L) was significantly more than the copper levels of the healthy volunteers (1.31 ± 0.24 mg/L; *p* = 0.048). Copper may have a role in the development of ISCMP.
Turkey, Atlihan et al. (1990)	29 patients (mean age 2.3 ± 1.5 years) with CHF and 11 healthy controls (mean age 3.1 ± 2.8 years)	The mean serum copper levels of the patients with CHF showed a significant increase compared to controls (173.6 ± 26.6 μg/100 mL vs. 113.9 ± 16.2 μg/100 mL, *p* ≤ 0.001)
Germany, Oster, (1993)	20 patients (mean age 50.5 ± 7.2 years) diagnosed with dilated cardiomyopathy and 50 healthy controls (mean age 53 ± 8 years)	The patients with dilated cardiomyopathy have high copper concentrations in their serum. The copper concentration in serum shows a highly significant inverse relationship with the ejection fraction and the cardiac index
Turkey, Koşar et al. (2006)	54 HF patients and 30 healthy subjects	Serum copper concentrations in HF patients were significantly higher than in controls (*p* = 0.000)
France, Cenac et al. (1996)	35 patients with peripartum cardiac failure and 40 healthy controls	Plasma copper was higher in patients with peripartum cardiomyopathy than it was in controls (2.03 ± 0.37 vs. 1.23 ± 0.20 μg/mL, *p* < 0.001)
Turkey, Topuzoglu et al. (2022)	54 patients (aged 18–75 years, the left ventricular ejection fraction <35%) with idiopathic dilated cardiomyopathy and 20 healthy subjects (aged 21–73 years)	Patients with idiopathic dilated cardiomyopaty have higher copper (172.16 ± 47.6 vs. 117.28 ± 31.3 μg/dL, *p* < 0.05)
France, de Lorgeril et al. (2001)	21 consecutive CHF patients and 18 healthy age- and sex-matched controls	Plasma copper was slightly higher in CHF than in healthy controls (1.15 ± 0.34 vs. 0.97 ± 0.17 mg/L, *p* < 0.05)
United State, Sullivan et al. (1979)	42 patients with arteriosclerotic congestive heart failure and 37 healthy controls	Copper levels were elevated in congestive heart failure (1.38 ± 0.33 vs. 1.06 ± 0.3 μg/mL, *p* < 0.005)

AHF, acute heart failure; CHF, chronic heart failure; AMI, acute myocardial infarction; ISCMP, ischemic cardiomyopathy.

Copper ions reportedly have dual effects on HF. A study in Poland reported serum copper deficiency in patients with HF (in 44% of men and >30% of women). Copper is an essential antioxidant nutrient for cardiovascular health (Mohammadifard et al., 2019), as evidenced by the benefits of copper supplementation and a copper-rich diet. The Institute of Medicine recommends a daily dietary copper intake of 0.9 mg for adults, with a tolerable upper limit of 10 mg/day to prevent liver toxicity (Trumbo et al., 2001). While national guidelines vary, most suggest a range of 0.8–2.4 mg/day (Bost et al., 2016). Evidence from a high-risk cross-sectional study involving 1,197 healthy adults revealed a negative correlation between dietary or serum copper levels and total/low-density lipoprotein cholesterol, suggesting that increased copper intake is associated with improved metabolic outcomes (Bo et al., 2008). Similarly, copper supplementation was shown to partially reverse pathological changes caused by dietary iron overload in mice, normalize cardiac hypertrophy (Wang et al., 2018), and enhance cardiac function in pressure overload-induced dilated cardiomyopathy (Hughes et al., 2008). HF patients often exhibit relative deficiencies in multiple micronutrients due to decreased intake, increased metabolic degradation, and excessive excretion, all of which exacerbate cardiac dysfunction (Witte and Clark, 2002). Supplementation with copper-containing micronutrients has been shown to improve the left ventricular ejection fraction, ventricular volume, and quality of life in elderly patients with left ventricular systolic dysfunction and HF (Witte et al., 2005). Cohort studies further suggest that dietary copper intake exceeding the estimated average requirement is associated with reduced cardiovascular and all-cause mortality, particularly when copper is obtained from food sources (Chen et al., 2004). Additionally, more than 80 anatomical, biochemical, and physiological parallels have been identified between copper-deficient animals and patients with ischemic heart disease (Klevay, 2006). In murine studies, dietary copper deficiency impaired β-adrenergic responsiveness and induced diastolic dysfunction, implicating copper insufficiency as a contributor to HF. These impairments were reversed with adequate copper consumption (Elsherif et al., 2003; Elsherif et al., 2004b; Liu and Miao, 2022). Collectively, these findings suggest that dietary copper supplementation may represent a cost-effective therapeutic strategy for managing HF.

Overall, the role of copper supplementation in the cardiovascular system remains unclear. Other studies have reported that serum copper levels do not significantly differ between patients with HF and control individuals (Cunha et al., 2002; Salehifar et al., 2008; Ghaemian et al., 2011). These inconsistent findings are likely associated with variations in study designs, the duration of copper supplementation, and the dosages used.

## 4 Potential mechanisms of HF associated with copper dyshomeostasis

Copper is involved in enzymatic activities, mitochondrial respiration, the maintenance of protein function, and iron metabolism (van den Berghe and Klomp, 2009; Dupuy et al., 2015; Niu et al., 2020; Guo et al., 2022b). The prevalence of copper excess in the human population actually surpasses that of copper deficiency, which is partly attributed to high inorganic copper ion consumption via mineral and vitamin supplements, as well as in drinking water from copper plumbing (Brewer et al., 2010; Pal et al., 2014). Hence, copper-mediated HF may be associated with several mechanisms described in this review.

### 4.1 Oxidative stress

**FIGURE 3:**
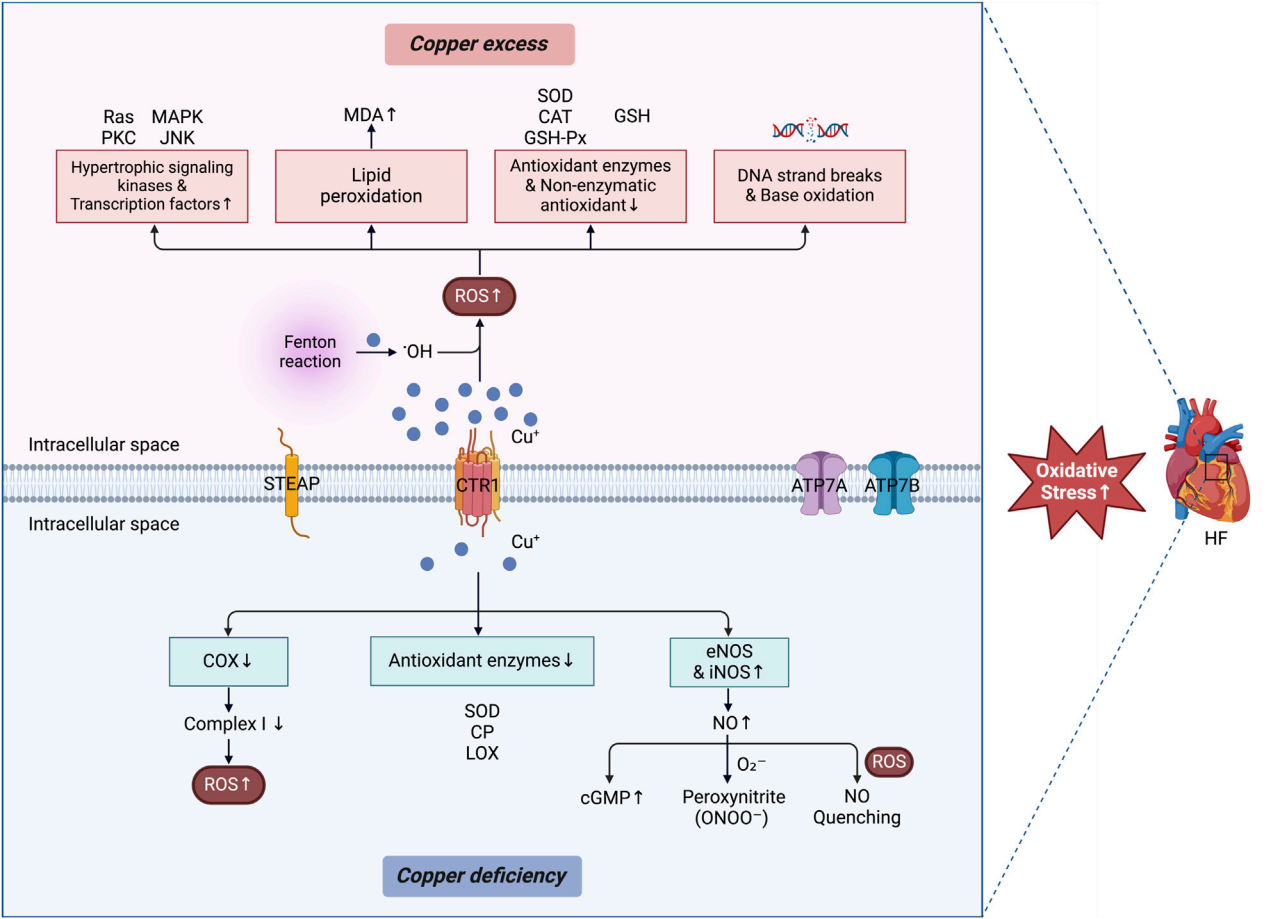
Copper dyshomeostasis and oxidative stress in HF. The oxidative stress mechanism associated with copper dyshomeostasis in HF has dual properties. Excess copper catalyzes the generation of •OH via Fenton reactions, resulting in the activation of hypertrophic signaling kinases and transcription factors, lipid peroxidation, DNA strand breaks, and base oxidation, while impairing the function of both enzymatic and nonenzymatic antioxidants. On the other hand, copper deficiency impairs the function of certain antioxidant enzymes. This leads to increased levels of NO and cGMP via elevated expression of eNOS and iNOS. Additionally, copper deficiency results in reduced COX activity, decreases complex I function and contributes to elevated ROS. Excess ROS directly quench bioavailable NO. Furthermore, O_2_– interacts with NO to form peroxynitrite (ONOO–). Together, these processes exacerbate intracellular oxidative stress and contribute to the progression of HF. The figure was created via BioRender. HF, heart failure; CTR1, copper transporter 1; STEAP, six-transmembrane epithelial antigen of the prostate; ATP7A and 7B, ATPase copper transporters 7A and 7B; •OH, hydroxyl radicals; ROS, reactive oxygen species; Ras, rat sarcoma; PKC, protein kinase C; MAPK, mitogen-activated protein kinase; JNK; Jun-nuclear kinase; MDA, malondialdehyde; SOD, superoxide dismutase; CAT, catalase; GSH-Px, glutathione peroxidase; GSH, glutathione; CP, ceruloplasmin; LOX, lysyl oxidase; eNOS, endothelial nitric oxide synthase; iNOS, inducible nitric oxide synthase; NO, nitric oxide; cGMP, cyclic guanosine monophosphate; COX, cytochrome c oxidase.

Oxidative stress plays a significant role in the occurrence and development of HF, typically resulting from an imbalance between ROS production and antioxidant defense mechanisms (Karabacak et al., 2014). Copper, a transition metal involved in redox reactions, facilitates ROS production, with prolonged exposure leading to oxidative stress (Vo et al., 2020) (Figure 3).

Higher levels of free copper ions may further interact with hydrogen peroxide through Fenton reactions, resulting in the generation of highly reactive hydroxyl radicals (Valko et al., 2005). These radicals induce cellular dysfunction, DNA damage, and lipid and protein peroxidation, ultimately promoting the initiation and progression (Tsutsui et al., 2011) of HF. Specifically, copper can activate various hypertrophic signaling kinases and transcription factors, including protein kinase C, GTP-binding protein Rat sarcoma, Jun N-terminal kinase, and mitogen-activated protein kinases (MAPK) (Grubman and White, 2014). This activation stimulates myocardial development, contributes to cellular dysfunction, and facilitates matrix remodeling. Lipid peroxidation is a chain reaction triggered by the accumulation of ROS in polyunsaturated fatty acids within cell membrane lipids, thereby resulting in lipid molecule oxidative damage. As an intracellular copper scavenger, MT has been demonstrated to be an endogenously expressed and highly inducible antioxidant protein in the heart. Yin et al. reported that mice with silenced MT1/2 genes developed severe HF, cardiac fibrosis, and oxidative stress, with these symptoms exacerbated by intermittent hypoxia. In contrast, mice exhibiting cardiac-specific overexpression of MT-IIa were protected from cardiomyopathy induced by intermittent hypoxia. This protective effect is associated with reduced cardiac lipid peroxidation in the context of copper deficiency (Yin et al., 2014). Furthermore, increased copper levels may increase lipid peroxidation, leading to increased generation of malondialdehyde (MDA) (Dhalla et al., 2000), whose high levels are associated with HF(Díaz-Vélez et al., 1996; Ghatak et al., 1996; Sairam et al., 2017). Additionally, increased copper levels can decrease the activities of antioxidant enzymes, including SOD, total antioxidant capacity, GSH peroxidase (GSH-Px), and catalase, in both serum and heart tissue while also lowering the levels of the nonenzymatic antioxidant GSH (Skrajnowska et al., 2013; Li et al., 2018). Blood GSH levels serve as an independent marker of lipid peroxidation in HF (Campolo et al., 2023). Copper can induce DNA strand breaks and base oxidation through free radicals generated from oxygen (Brezová et al., 2007). Existing evidence suggests that copper-induced oxidative stress significantly impacts HF.

### 4.2 Inflammation

Excessive copper is been related to the pathogenic mechanisms of HF because it can cause inflammation. Since its first discovery by Levine et al. (1990), higher circulating levels of proinflammatory factors have been associated with poor HF outcomes (Aukrust et al., 2002; Heymans et al., 2009; Hansson and Hermansson, 2011). Reportedly, increased copper levels can stimulate increases in the serum levels of tumor necrosis factor α (TNF-α) and C-reactive protein in rats, contributing to inflammatory damage in multiple organs, including the heart, and inducing myocardial fibrosis (Wang et al., 2022). *In vitro* experiments revealed that copper enhances interleukin (IL)-6 release while activating MAPK extracellular signal-regulated kinase 1/2 and p38 in primary cardiac cells (Ansteinsson et al., 2009), which are associated with mechanisms of cardiac hypertrophy, an important risk factor for the development of HF(Bueno and Molkentin, 2002; Wenzel et al., 2005). Copper-mediated oxidative stress further exacerbates inflammation, as excess copper leads to excessive ROS accumulation, which then increases myeloperoxidase activity, activates the nuclear factor-κB (NF-κB) pathway, inhibits anti-inflammatory cytokines, and leads to a proinflammatory environment (Pereira et al., 2016; Jian et al., 2020).

Additionally, the molecular interplay between copper homeostasis and immune signaling pathways has revealed novel mechanisms by which copper drives excessive and deleterious inflammation during disease progression. Copper uptake, which is mediated by CD44, regulates immune cell activation; however, dysregulated activation triggers uncontrolled inflammation, leading to tissue damage and organ failure, with copper directly acting as a catalytic metal (Solier et al., 2023). Furthermore, copper has been shown to activate the innate immune pathway through ALPK1 in a kinase-dependent manner, amplifying downstream signaling and increasing proinflammatory cytokine production (Lu et al., 2024). Although autoimmunity is not a predominant driver of cardiac dysfunction, it remains a potential target for HF prevention (Martini et al., 2024).

### 4.3 Energy metabolism dysfunction

Patients with HF typically exhibit features of inadequate cardiac energy metabolism, primarily because of reduced mitochondrial oxidative capacity (Lopaschuk et al., 2021). Copper serves as a cofactor for several enzymes involved in the antioxidant system and the mitochondrial respiratory chain (Nair and Mason, 1967). However, copper accumulation negatively affects mitochondrial activity and may contribute to HF progression by reducing mitochondrial metabolism (Fan et al., 2005; Rines and Ardehali, 2013). For example, increased copper levels can lead to excessive ROS generation, which facilitates lipid peroxidation, depletes antioxidants, damages mitochondrial DNA, and reduces mitochondrial ATP production (Sugamura and Keaney, 2011; Tsutsui et al., 2011). Furthermore, the failing heart also exhibits a marked decrease in key enzymatic activities, such as mitochondrial creatine kinase (CKmito), which operates at the intersection of energy metabolism and oxidative stress (Keceli et al., 2022). CKmito dysfunction disrupts efficient energy transfer between mitochondria and myofibrils, exacerbating energy deprivation in cardiomyocytes (Keceli et al., 2022). Mitochondrial ATP is primarily generated through fatty acid oxidation, which serves as the heart’s main fuel source (Yamamoto and Sano, 2022). Copper can also directly impair enzymes associated with the mitochondrial oxidative phosphorylation (OXPHOS) chain and fatty acid β-oxidation, resulting in mitochondrial insufficiency and energy starvation (Mazi et al., 2020). This cascade of events ultimately contributes to HF progression.

### 4.4 Cell death

High copper levels are associated with cell death, such as pyroptosis, apoptosis, ferroptosis, and autophagy (Jiang et al., 2022).

#### 4.4.1 Apoptosis

Apoptosis is a programmed cell death process that plays a crucial role in the progression of HF(Vanempel et al., 2005). Copper can induce apoptosis primarily by causing DNA damage, generating ROS, and suppressing proteasome activity (Sagripanti et al., 1991; Kawakami et al., 2008; Chen X. et al., 2021). The apoptosis of myocardial cells, which are the contractile units of myocardial tissue, triggers various reactions, such as fibrosis and hypertrophy. These reactions ultimately lead to both contractile and diastolic dysfunction, contributing to the progression of HF(Vanempel et al., 2005).

#### 4.4.2 Pyroptosis

Pyroptosis is a proinflammatory form of programmed cell death resulting from the activation of caspase family proteins (Kovacs and Miao, 2017; Wu et al., 2021). The increased cellular uptake of copper, combined with increased Fenton activity, can significantly increase intracellular ROS levels, thereby activating Caspase-1 proteins and pyroptosis-related genes, such as nucleotide-binding oligomerization domain-like receptor protein 3 inflammasomes and IL-1β. This activation subsequently leads to gasdermin D cleavage, ultimately inducing pyroptosis (Jiang K. et al., 2007; Liao et al., 2019; Zhang et al., 2021). Moreover, copper ions, as essential trace nutrients involved in various physiological processes, can induce ROS and activate the NF-κB pathway. This process upregulates the expression of proinflammatory factors and genes, which may contribute to the development of HF(Butts et al., 2015; Liu et al., 2020; Deng et al., 2023).

#### 4.4.3 Autophagy

Copper ions can trigger autophagy (Tsang et al., 2020). Mechanistically, increased copper levels produce excessive ROS during oxidation, which is associated with the occurrence of autophagy (Li et al., 2015). Additionally, excessive copper may increase autophagic flux through the activation of the Beclin-1 (Wan et al., 2020), autophagy-related gene 5 (Wan et al., 2020), and the adenosine monophosphate-activated protein kinase-mammalian target of rapamycin (Guo et al., 2022a) pathways, promoting the generation of autophagic vesicles by regulating the transcription factor EB (Peña and Kiselyov, 2015). Autophagy has dual functions. Excessive mitophagy and autophagy in various HF models are recognized as protective responses of cardiomyocytes (Shires and Gustafsson, 2015). However, some researchers have suggested that impaired mitophagy can lead to unfavorable myocardial pathological remodeling. The activation of mitochondrial autophagy can cause the heart to transition from adaptive compensatory hypertrophy to myocardial fibrosis, ultimately progressing to HF(Zhu et al., 2007; Li A. et al., 2022). These outcomes may be influenced by the strength of the stimulus and the type of substrate targeted for degradation (Xue et al., 2023a).

#### 4.4.4 Ferroptosis

Ferroptosis refers to iron-dependent cell death resulting from ROS deposition and lipid peroxidation (Dixon et al., 2012; Yang and Stockwell, 2016; Hirschhorn and Stockwell, 2019) and is affected by copper content because copper ions are redox active (Ren et al., 2021; Zhou et al., 2023). GSH-Px 4 (GPX4) is a crucial gene associated with ferroptosis. Copper can directly bind to the GPX4 protein, leading to the formation of GPX4 aggregates and subsequent autophagic degradation of GPX4, ultimately resulting in ferroptosis (Xue et al., 2023b). Copper chelators may reduce sensitivity to ferroptosis without affecting other types of cell death. Fisetin has cardioprotective effects; it modulates GPX4 expression to increase its antioxidant activity and suppress ferroptosis, ultimately ameliorating cardiac hypertrophy (Li et al., 2016).

Additionally, agents that bind to copper, along with their corresponding copper complexes, such as elesclomol-copper and disulfiram-copper, can disrupt mitochondrial homeostasis and induce oxidative stress, ultimately leading to ferroptosis in cancer cells (Wollert et al., 2002; Gao et al., 2021). These findings align with evidence that excess copper deposition generates excessive ROS within cardiomyocytes (Huang et al., 2024).

### 4.5 Dual effects of copper dyshomeostasis in the induction of HF

Copper deficiency contributes to the occurrence and progression of HF through multiple mechanisms, particularly its impact on oxidative stress. Copper is an essential cofactor for several antioxidant enzymes, including Cu/Zn superoxide dismutase (Cu/Zn SOD), CP, and LOX (Al-Aubaidy et al., 2015). Consequently, copper deficiency can impair the activity of these enzymes, leading to a compromised antioxidant defense system and increased susceptibility to oxidative stress (Chen et al., 1994; Lynch and Colón, 2006; DiNicolantonio et al., 2018). In addition to its effects on antioxidant enzymes, copper deficiency can decrease COX activity, leading to the oxidative inactivation of complex I (NADH:ubiquinone oxidoreductase). This process can subsequently increase ROS production in copper-deficient cells, thereby exacerbating oxidative stress (Johnson and Thomas, 1999). Copper supplementation has been shown to restore COX activity, resulting in the reversal of myocardial hypertrophy (Jiang Y. et al., 2007). Copper deficiency also disrupts NO homeostasis, particularly by affecting Cu/Zn SOD activity in endothelial cells (Lynch et al., 1997). NO is a crucial vasodilator that plays an essential role in maintaining cardiovascular health. Reduced copper levels lower NO production, leading to endothelial dysfunction, impaired vasodilation, increased oxidative stress, and vascular complications associated with HF(Al-Aubaidy et al., 2015). Interestingly, copper deficiency has a different effect on NO generation in the heart than in endothelial cells. For example, Sarri et al. reported that copper deficiency promoted NO production in the rat heart by increasing the expression of inducible NO synthase and endothelial NO synthase proteins (Saari and Dahlen, 1998; Saari, 2000; Saari et al., 2007). While NO can facilitate HF through its interaction with superoxide to form peroxynitrite, a reactive and long-lived radical that amplifies oxidative stress (Kim et al., 1999; Wollert and Drexler, 2002), excess ROS can also directly quench bioavailable NO. As noted by Paolocci et al., this reduction in NO bioavailability impairs its vasorelaxant capacity and contributes to endothelial dysfunction (Paolocci et al., 2001). The balance between these two effects—NO consumption through peroxynitrite formation and reduced NO availability due to ROS quenching—underpins the endothelial dysfunction and vascular complications observed in HF in the context of copper deficiency. Furthermore, copper deficiency increases cyclic guanosine monophosphate levels, which can impair cardiac contractility and exacerbate HF progression in animal models (Saari and Dahlen, 1998; Saari, 2000; Saari et al., 2007). Collectively, these findings underscore the complex role of NO in the pathophysiology of heart disease associated with copper deficiency. However, further studies are needed to elucidate the molecular mechanisms by which NO regulates cardiac pathologies in the context of copper deficiency.

Decreased responsiveness to β-adrenergic stimulation is a hallmark of copper deficiency-induced HF, as evidenced by the reduced sensitivity of copper-deficient mouse hearts to the β-adrenergic agonist isoproterenol (Elsherif et al., 2003). β-Adrenergic receptors, members of the G-protein-coupled receptor family, are regulated through changes in expression and function in response to external stimuli, resulting in alterations in heart rate, contractility, relaxation, and automaticity (Pfleger et al., 2019). Dysregulation of β-adrenergic receptor signaling has been associated with congestive heart failure caused by cardiomyopathy. Proposed mechanisms include reduced receptor expression, downregulation of G-proteins, or impaired adenylate cyclase activity due to phosphorylation (Elsherif et al., 2003). The impaired adrenergic responsiveness observed in copper-deficient hearts raises questions about its potential relationship with altered catecholamine metabolism under copper-deficient conditions (Gross and Prohaska, 1990; Prohaska et al., 1990; Seidel et al., 1991). Notably, functional changes have been shown to precede structural damage in copper deficiency. In rats subjected to a copper-restricted diet for 9 or 15 months, both diastolic and systolic dysfunctions were observed. This was indicated by a blunted response in the maximal left ventricular pressure elevation rate (+dP/dt), the maximal left ventricular pressure decrease rate (-dP/dt), and the left ventricular end-diastolic pressure in response to isoproterenol (Li et al., 2005). In a previous study, feeding copper-deficient mice a diet adequate in copper for 4 weeks completely restored cardiac systolic and diastolic functions, as well as responsiveness to β-adrenergic stimulation (Elsherif et al., 2004b). These findings indicate that the response of the heart to β-adrenergic stimulation is dependent on copper levels.

The molecular mechanisms underlying HF induced by copper deficiency involve disrupted cellular calcium homeostasis. Altered myocardial contractility during end-stage HF is associated with changes in Ca^2+^ cycling (Hasenfuss et al., 1996; Hasenfuss and Pieske, 2002). This homeostasis is regulated primarily by sarcoplasmic/endoplasmic reticulum Ca^2+^-ATPase (SERCA), ryanodine receptors (RyRs), and the sodium/calcium exchanger (NCX) (Bers, 2002). Kang et al. reported that a copper-deficient diet markedly altered the expression of calcium cycling genes in the mouse heart, including a reduction in L-type calcium channels, which affected calcium release from the sarcoplasmic reticulum via potassium-dependent NCX and RyRs. Although there is a lack of cardiac functional data, the expression of these calcium-regulating genes was notably normalized in mice with copper deficiency following supplementation with a copper-replete diet (Elsherif et al., 2004a). Additionally, copper deficiency may impair calcium homeostasis and cardiac contractile activity by increasing phospholamban levels, which inhibits SERCA2a-mediated calcium uptake (Elsherif et al., 2007).

Additionally, copper deficiency has been reported to lead to alterations in myocardial gene expression in mice, particularly in genes involved in cardiac contractility, fibrosis, and inflammation. These changes are potential factors contributing to the alterations in cardiac activity observed in mice with copper deficiency (Elsherif et al., 2007). Further studies are essential to explore the underlying mechanisms of these findings.

## 5 Copper-mediated cell death and HF

### 5.1 Copper-mediated cell death and cuproptosis

Copper-mediated cell death was first discovered in the late 1970s when Chan et al. identified mechanisms regulating copper levels within healthy fibroblasts, noting that increased copper levels resulted in cell death (Chan et al., 1978). However, the precise mechanisms remain unknown. Since then, the copper-mediated cell death mechanism has garnered significant attention from researchers, and copper has been found to have dual effects. Over the last decade, the toxicity of essential trace metals to mammalian cells has become increasingly understood. In a recent study, such metals were found to induce cell death through mechanisms independent of established pathways, such as apoptosis or necrosis, as observed with zinc and iron (Dineley et al., 2003; Kagan et al., 2017; Du et al., 2021). This observation may also signal the gradual emergence of a mechanism for a noncanonical copper-induced cell death pathway.

In March 2022, Tsvetkov et al. published a groundbreaking paper in *Science* unveiling cuproptosis, a unique form of regulated cell death resulting from mitochondrial copper accumulation (Tsvetkov et al., 2022); they reported that copper accumulation in cells was a major factor triggering cuproptosis. Notably, after copper ionophores induce cell death, traditional markers of cell death, such as caspase-3, are not detected. Furthermore, only copper chelators can rescue cells from elesclomol-induced death. Inhibiting established cell death pathways—such as apoptosis, necroptosis, oxidative stress, and ferroptosis—fails to prevent cell death, highlighting the distinct nature of cuproptosis.

**FIGURE 4:**
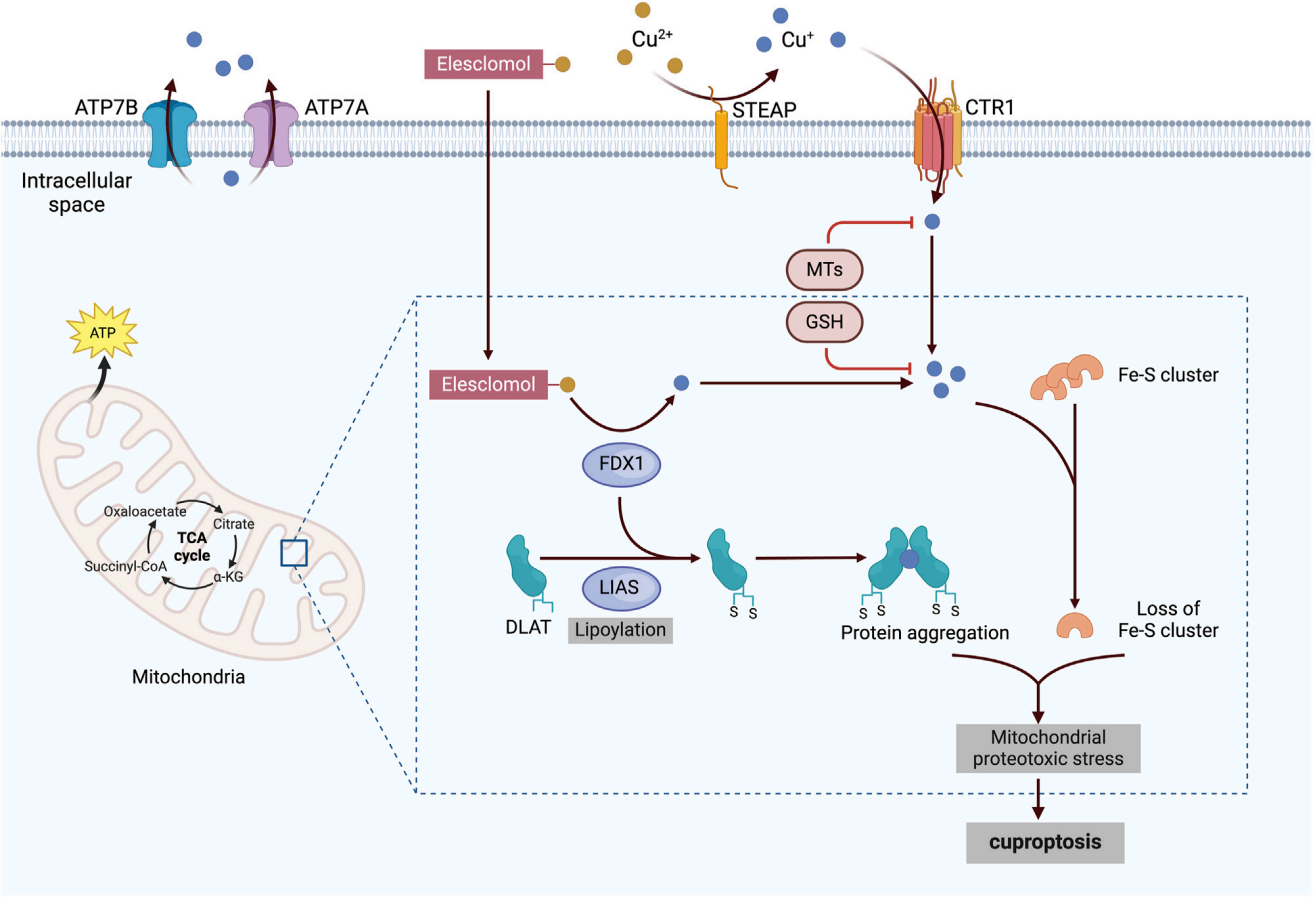
Cuproptosis mechanisms and mitochondrial dysfunction. Copper ionophores, such as elesclomol, bind extracellular copper and transport it into the cell, where it interacts with lipoylated enzymes of the TCA cycle, such as DLAT. FDX1 and LIAS serve as critical upstream regulators of this process, promoting protein aggregation and Fe-S cluster inhibition. Together, these events induce mitochondrial proteotoxic stress, ultimately leading to cuproptosis. The figure was created via BioRender. CTR1, copper transporter 1; STEAP, six-transmembrane epithelial antigen of the prostate; ATP7A and 7B, ATPase copper transporters 7A and 7B; α–KG, α–ketoglutarate; FDX1, ferredoxin–1; LIAS, lipoyl synthase; DLAT, dihydrolipoamide S-acetyltransferase; Fe–S, iron–sulfur; MT, metallothionein; GSH, glutathione.

Tsvetkov et al. reported that copper ionophore-induced cell death is dependent on mitochondrial respiration, as evidenced by the heightened sensitivity of mitochondria-dependent cells to copper ionophores, which are 1,000 times more sensitive than glycolytic cells (Tsvetkov et al., 2022). Further investigations revealed a time-dependent increase of tricarboxylic acid (TCA) cycle metabolite levels in cells exposed to copper ionophores, underscoring the close relationship between cuproptosis and the TCA cycle (Li Y. et al., 2022). In cuproptosis, copper in cells can bind to lipoylated components of the TCA cycle, resulting in the aggregation of copper-bound lipoylated mitochondrial proteins. This aggregation disrupts the TCA cycle, thereby impairing cellular energy generation. Ferredoxin and lipoyl synthase, two upstream regulatory factors, play significant roles in this process and have been identified as key contributors to copper toxicity through whole-genome clustered regularly interspaced short palindromic repeat selection (Dreishpoon et al., 2023). The aggregation of proteins and the subsequent decrease in iron‒sulfur clusters—important cofactors for various cellular processes, such as enzymatic reactions and electron transport (Lill and Freibert, 2020)—promote toxic protein stress, ultimately leading to cell death (Figure 4).

### 5.2 Cuproptosis-associated mitochondrial dysfunction and HF

Mitochondria serve as significant reservoirs of intracellular copper, which is a critical component that regulates mitochondrial function. Hence, pathologies and diseases resulting from copper metabolism disorders are closely linked to mitochondrial metabolism (Tian et al., 2023). Mitochondria are highly vulnerable to copper-mediated injury, leading to oxidative damage to their membranes (Arciello et al., 2005; Cobine et al., 2021).

Copper serves as a cofactor for various mitochondrial enzymes involved in ATP production, a process that relies on the TCA cycle and OXPHOS within mitochondria (Arnold and Finley, 2023). However, increased copper content in cells can impair mitochondrial activity by altering critical enzymes associated with these processes (Sheline and Choi, 2004; Wang Z. et al., 2023). This disruption also generates ROS, which subsequently damage the inner mitochondrial membrane, impair the electron transport chain, and compromise mitochondrial DNA. This damage leads to mitochondrial dysfunction and reduced ATP production, ultimately accelerating cell death (Tsutsui et al., 2011).

Furthermore, metabolic enzymes may undergo lipoylation, a conserved posttranslational modification (Rowland et al., 2018). Lipoylation has been identified in glycine cleavage system protein H, dihydrolipoamide branched-chain transacylase E2, dihydrolipoamide S-acetyltransferase, and dihydrolipoamide S-succinyltransferase, all of which are associated with metabolic complexes that regulate the entry points of carbon into the TCA cycle (Rowland et al., 2018; Solmonson and DeBerardinis, 2018). Lipoylation involves the attachment of lipoic acid, a small sulfur-containing metabolite, to substrate proteins. Notably, lipoic acid can bind copper, leading to the toxic accumulation of lipoylated mitochondrial enzymes within the mitochondria (Kahlson and Dixon, 2022).

Moreover, excessive copper concentrations within the mitochondria can compromise mitochondrial membrane integrity by disrupting the membrane potential and increasing membrane permeability (Gyulkhandanyan et al., 2003; Reddy et al., 2008a). The opening of transmembrane pores, a critical factor in mitochondrial permeability transition, is not fully understood in terms of its precise chemical nature, but it is likely associated with the release of necrotic or apoptotic factors (Zazueta et al., 1998; Su et al., 2011). Additionally, the strong oxidizing nature of Cu(I) further contributes to potentially irreversible damage to the mitochondrial membrane (Reddy et al., 2008b).

The heart is the central organ for energy production in the human body, with about 95% of its ATP generated through oxidative metabolism in the mitochondria (Zhou and Tian, 2018). Consequently, mitochondrial dysfunction is linked to the onset and progression of various cardiovascular diseases. Mitochondrial dysfunction has been reported to affect cardiac energy supply, inflammatory mechanisms, calcium modulation, oxidative stress, and cell death, all of which are critical therapeutic targets for HF(Hammadah et al., 2014; Zhang et al., 2020; Yuan et al., 2022). In studies involving rats with HF, copper chelation and restoration within cardiomyocytes have been shown to repair mitochondria while improving cardiac function (Zhang et al., 2020). Hence, cuproptosis represents a novel mechanism for treating HF.

## 6 Possible treatments for cuproptosis in HF

Copper-based drugs have been extensively studied not only for cancer treatment but also for their multifaceted impact on HF therapy (Chen et al., 2011; Wang et al., 2024). These agents have promising effects in the treatment of HF from various perspectives. Copper-based therapies may enhance HF management by providing individualized, targeted, and efficient treatment methods.

### 6.1 Copper chelators

As a chelator, triethylenetetramine (TETA) specifically and selectively binds to Cu(II) and is used as a second-line therapy for Wilson’s disease (Walshe, 1982; Cooper, 2011). TETA treatment can enhance the regeneration of cardiac structure and function in HF model mice with diabetes (Cooper et al., 2004; Zhang et al., 2007; 2014; Nurchi et al., 2016) and reduce pathological left ventricular hypertrophy in patients with diabetes (Cooper et al., 2004; 2009). Moreover, TETA can restore cardiac pump activity by enhancing the activities of mitochondrial proteins such as COX, mitochondrial CCS, and SOD1, as well as by reinstating the expression of peroxisome proliferator-activated receptor gamma coactivator-1α, an important regulatory factor for mitochondrial biogenesis (Zhang et al., 2020). TETA has reportedly advanced to drug development for treating HF (Cooper G, 2012).

Owing to its copper-chelating properties, tetrathiomolybdate (TTM), which was initially developed for treating Wilson’s disease, has been explored for its potential therapeutic benefits in HF(Pufahl et al., 1997; Alvarez et al., 2010), which effectively prevents copper transport and its subsequent incorporation into cuproproteins. Although the effects of TTM on cardiac conditions remain elusive, some studies have highlighted its ability to reduce systemic copper overload and its associated proinflammatory consequences, which are relevant in the context of HF. For example, Wei et al. reported that TTM suppresses the expression of NF-κB, TNF-α, and monocyte chemoattractant protein-1 in the aorta and heart (Wei et al., 2011), which play crucial roles in inflammation. These findings open avenues for exploring the role of TMM in HF treatment, indicating the need for further research to directly assess its efficacy in treating cardiac diseases.

### 6.2 Small-molecule inhibitors of copper chaperone proteins

Copper ion chelation therapies can reduce copper levels; however, they lead to various side effects and disrupt various copper-dependent physiological processes (Chen et al., 2011). On the other hand, while copper supplementation can address copper deficiency, it carries the risk of copper overload. Excessive copper levels can exacerbate oxidative stress through the generation of ROS, impair mitochondrial function, and disrupt cellular homeostasis, particularly in the heart and liver (Kaplan and Maryon, 2016). These challenges highlight the need for more targeted approaches to modulate copper homeostasis in HF therapy. To minimize these adverse effects, small-molecule inhibitors of copper chaperone proteins offer a promising alternative. Unlike chelation or supplementation strategies, these inhibitors focus on the selective redistribution and regulation of intracellular copper levels, thereby reducing the risks associated with copper imbalance.

DCAC50 is a promising small-molecule inhibitor that selectively disrupts the functions of copper chaperones by binding to proteins such as CCS and ATOX1 (Karginova et al., 2019). ATOX1 is involved in transporting copper to the cytosol, whereas CCS facilitates the delivery of copper to SOD1 (Rosenzweig and O'Halloran, 2000). In studies of atherosclerosis, ATOX1 is increased in the intima of atherosclerotic lesions in ApoE^−/−^ mice and is localized to the nucleus under pathological conditions, including hypertensive and atherosclerotic vessels (Jeney et al., 2005; Itoh et al., 2008). Similarly, CCS plays a vital role in angiogenesis and wound healing, with its ability to impair these processes and contribute to the progression of cardiovascular diseases (Fukai et al., 2018). While both ATOX1 and CCS are critical for normal physiology, their dysregulation under pathological conditions underscores the potential for selectively targeting copper chaperones as a therapeutic strategy. Given the pivotal role of copper homeostasis in maintaining cardiac function, DCAC50 offers a targeted approach to modulate copper chaperone activity while preserving physiological copper-dependent processes. Further studies on small-molecule inhibitors of copper chaperone proteins are needed to develop effective treatments for HF.

### 6.3 Copper ionophores

Copper ionophores represent another therapeutic strategy for modulating copper levels and influencing related cellular pathways in HF. Unlike copper chelators, which remove copper from cells, copper ionophores facilitate the delivery of copper into cells, thereby increasing the intracellular copper concentration. Some examples of these ionophores include pyrithione, disulfiram, elesclomol, and chloroquine (Xue et al., 2023a). Among them, elesclomol has garnered considerable attention owing to its sensitivity to tumor cells and its application in clinical studies for cancer therapy (O’Day et al., 2013). However, the mechanisms underlying its selectivity remain unelucidated, warranting further investigation to determine whether this selectivity can be adapted for treating HF with additional copper ionophores. Su et al. incorporated metal supplements via a targeted ion carrier approach designed to deliver metals to specific sites within the body (Su et al., 2018). This strategy addresses the limitations of conventional copper ionophores, particularly their multifunctionality and lack of specificity, thereby opening new avenues for applying copper in HF treatment. Additionally, nanomedicine-based drug delivery systems are being widely explored to enable the precise delivery of therapeutic agents (Su et al., 2018). These developments underscore the need for future research focused on developing more selective and targeted copper ionophores for HF therapy.

## 7 Conclusion and future perspectives

Overall, the diverse effects of copper on HF are linked to complex systemic and cellular metabolism. Copper has dual effects on HF, and excessive or deficient copper can contribute to disease progression by regulating oxidative stress, inflammation, energy metabolism, cell death, the response to β-adrenergic stimulation, and calcium homeostasis. Further investigations are warranted to elucidate the interactions among these factors and their effects on HF progression.

Copper-mediated cell death and the subsequent process of cuproptosis can increase our understanding of the effects of copper on HF. The relationship between cuproptosis and mitochondrial dysfunction in HF underscores the necessity of exploring the molecular mechanisms associated with these processes. The development of copper-based therapies that target cuproptosis is a promising approach and has therapeutic potential for the treatment of not only cancer but also cardiovascular diseases. However, further investigations are needed to determine the role of cuproptosis in causing cell injury and to identify reliable specific biomarkers, which can provide crucial insights for the prevention and management of HF.
